# Supplementary material for: Stochasticity in Dietary Restriction-Mediated Lifespan Outcomes in Drosophila
Source: Res Sq. 2024 Sep 17:rs.3.rs-4876799. Preprint. [Version 1] doi: 10.21203/rs.3.rs-4876799/v1 (PMC11451724; doi:10.21203/rs.3.rs-4876799/v1)
Supplement: Supplement 1 [file NIHPPRS4876799V1-supplement-1.pdf]

Supplemental Figure legends

Supplemental Figure 1. Kaplan-Meier curves of each of 64 pairs of AL/DR experiments.

Supplemental Figure 2. Difference of AL and DR median lifespan between labs.

Supplemental Figure 3. Climbing results for 30-day old flies from the Hoffman lab for females (A) and males (B). Each replicate consists of 18 vials of ~20 flies each. Mean climbing values were taken on a per vial average. Cohorts 1-3 were combined for analysis. There were significant effects of sex and genotype with no difference between AL and DR treatments.

Supplemental Figure 4. Body mass results for 30-day old flies on SY5 vs SY15 for females (A) and males (B). Each replicate consisted of ~ 5 measurements of 5 flies each. There were no significant effects of treatment, suggesting that our flies were not calorically restriction on the DR treatment. Females were significantly larger than males as expected, and no genotype effects were seen.

Supplementary Files

This is a list of supplementary files associated with this preprint. Click to download.

- SupplementaryFigure1Lifespan.pdf
- SupplementaryFigure2ALDRdifferencesacrosslabs.pdf
- SupplementaryFigure3Climbing.pdf
- SupplementaryFigure4Weights.pdf
- SupplementaryTable1RawData.csv
- SupplementaryTable2CoxSnellR2.pdf
